# Radiological assessment of hip disease in children with cerebral palsy: development of a core measurement set

**DOI:** 10.1302/2633-1462.411.BJO-2023-0060.R1

**Published:** 2023-11-01

**Authors:** Prince J. S. Joseph, Mohammed Khattak, Sundas T. Masudi, Louise Minta, Daniel C. Perry

**Affiliations:** 1 University of Liverpool, Liverpool, UK; 2 Alder Hey Children’s Hospital, Liverpool, UK; 3 Hull University Teaching Hospitals, Hull, UK

**Keywords:** hip, hip migration, hip measurements, radiographic measurements, cerebral palsy, core measurement set, Cerebral palsy, hip disease, hips, consultant orthopaedic surgeons, femoral head, clinicians, physiotherapists, Delphi process, radiological outcomes, clinical studies

## Abstract

**Aims:**

Hip disease is common in children with cerebral palsy (CP) and can decrease quality of life and function. Surveillance programmes exist to improve outcomes by treating hip disease at an early stage using radiological surveillance. However, studies and surveillance programmes report different radiological outcomes, making it difficult to compare. We aimed to identify the most important radiological measurements and develop a core measurement set (CMS) for clinical practice, research, and surveillance programmes.

**Methods:**

A systematic review identified a list of measurements previously used in studies reporting radiological hip outcomes in children with CP. These measurements informed a two-round Delphi study, conducted among orthopaedic surgeons and specialist physiotherapists. Participants rated each measurement on a nine-point Likert scale (‘not important’ to ‘critically important’). A consensus meeting was held to finalize the CMS.

**Results:**

Overall, 14 distinct measurements were identified in the systematic review, with Reimer’s migration percentage being the most frequently reported. These measurements were presented over the two rounds of the Delphi process, along with two additional measurements that were suggested by participants. Ultimately, two measurements, Reimer’s migration percentage and femoral head-shaft angle, were included in the CMS.

**Conclusion:**

This use of a minimum standardized set of measurements has the potential to encourage uniformity across hip surveillance programmes, and may streamline the development of tools, such as artificial intelligence systems to automate the analysis in surveillance programmes. This core set should be the minimum requirement in clinical studies, allowing clinicians to add to this as needed, which will facilitate comparisons to be drawn between studies and future meta-analyses.

Cite this article: *Bone Jt Open* 2023;4(11):825–831.

## Introduction

Cerebral palsy (CP) is the most common childhood physical disability.^1^ Individuals with CP are at a high risk of developing hip disease, which can cause pain, decrease quality of life, and decrease function.^2,3^ Adduction contractures associated with hip disease can worsen sitting balance and make caring for children with CP problematic.^4^ Once hips begin to laterally displace, they are unlikely to resolve spontaneously, and, left untreated, often progress to complete dislocation.^5^ Subluxed hips are more readily treated if detected early.^6^ National hip surveillance programmes have been implemented in many countries to detect hip displacement earlier and to enable timely intervention.^7-12^ Several studies have reported a significant decrease in the incidence of hip dislocation in regions where a hip surveillance programme has been introduced.^6,11,13^

Currently, radiological hip analysis in surveillance programmes, such as the Cerebral Palsy Integrated Pathway Scotland (CPIPS), is performed manually by experts.^14^ Radiograph analysis is time-consuming both to perform and upload data, and is prone to variability between clinicians.^15^ Tools within Picture Archiving and Communication Systems (PACS) or mobile apps may have accelerated this process;^16,17^ however, a fully-automated system is not currently available.

Artificial intelligence (AI) can be used to optimize image interpretation.^18^ The development of AI software capable of automating radiological hip analysis in CP firstly requires the essential measurements to be clearly defined; the core measurement set (CMS). The CMS has the potential to be a minimum data requirement which would standardize the measurements used, enabling effective comparisons to be drawn in clinical research.

This study aimed to form a CMS containing the universal measurements necessary to record in the assessment of CP hip radiographs. The COMET (Core Outcome Measures in Effectiveness Trials) guidelines were adapted,^19^ and used as a guide to form the CMS.

## Methods

A systematic review was used to identify previously reported radiological measurements of CP-related hip disease and inform a Delphi process to generate consensus.

### Systematic review

This review was conducted as per the Preferred Reporting Items for Systematic Reviews and Meta-Analyses (PRISMA) guidelines,^20^ and aimed to identified all the radiological measurements reported in the literature. The electronic databases searched included PubMed, SCOPUS, and Web of Science (Supplementary table i).

### Eligibility criteria

The inclusion criteria comprised studies that used radiological measurements to assess pelvic radiographs in CP hip disease, with ≥ 50 participants, participants aged ≤ 18 years, English language, publication after first January 2011, and studies that were case series, cross-sectional studies, cohort studies, or randomized control studies. Studies were excluded if they primarily concerned measurement reliability, or the full-text was not available.

### Study selection and data extraction

Articles were downloaded and duplicates excluded. The remaining articles’ abstracts were screened independently by two reviewers (PJSJ, STM) according to the inclusion/exclusion criteria. Disagreements were resolved via discussion. Articles were then reviewed in the full-text format to confirm eligibility. Disagreements during the full-text analysis were resolved by consulting a third review author (MK).

The following data fields were independently extracted from each article by two reviewers: title, author, year, journal of publication, location, study type, population size, number of hips studied, age, sex, duration of follow-up, measurement used, time point of measurement, verbatim definition of measurement, use of visual explanations for measurements, and primary intervention. A study was considered to have defined a measurement if it provided a definition or a visual explanation.

Radiological measurements were collated irrespective of study quality; therefore, an appraisal of the methodological quality of the studies or risk of bias assessment was not undertaken.

### Delphi study

### Overview

The modified Delphi method was used to seek consensus regarding the radiological measurements forming the CMS. This consisted of two stages: 1) a two-round Delphi survey to score the identified measurements on importance; and 2) a final consensus meeting to establish the CMS.

The Delphi survey (Supplementary figure a) was initiated using the list of radiological measurements identified from the systematic review.

### Participants and process

The Delphi study was conducted among orthopaedic surgeons and physiotherapists with a specialist interest in CP. Participants from any country were eligible to take part, and were recruited via the investigators' networks and newsletters disseminated by the British Society for Children’s Orthopaedic Surgery (BSCOS).^21^ A total of 20 participants were sought to take part as a minimum.

Participants were given four weeks to complete each round. Reminder emails were sent during weeks two and three if a participant failed to complete the online questionnaire. Failure to complete the questionnaire before the deadline resulted in exclusion from further rounds.

### Scoring and defining consensus

Each radiological measurement was scored using the GRADE (Grading of Recommendations, Assessment, Development and Evaluations) approach.^22^ A nine-point Likert scale was used, with 1 to 3 considered 'not important', 4 to 6 considered 'important but not critical', and 7 to 9 considered 'critically important'.

Consensus definitions were pre-defined to avoid bias and based on the '70/15%' consensus framework described in the COMET handbook version 1.0.^19^ Inclusion required the majority of participants (> 70%) to score the measurement in question as being 'critically important', with only a small minority (< 15%) considering it to be 'not important'; exclusion was the opposite. Measurements that did not reach the consensus threshold were considered equivocal and were discussed in a final consensus meeting.

In round one, participants were asked to score the measurements, and suggest any additional measurements that the respondents considered important. Measurements not reaching consensus in round one, and additionally added measurements, were taken to round two. In round two, participants were presented with the results from round one and asked to score the measurements.

### Consensus meeting

A final consensus setting exercise was conducted online in May 2022. A small, focused group was selected to facilitate meaningful and efficient discussions and decision-making regarding the equivocal measurements. Five participants attended the meeting: four consultant orthopaedic surgeons (MG, SC, DMW, DCP), chosen for their expertise in CP and hip surveillance, and an independent chair, who did not partake in the consensus discussion; the final CMS was established at this stage.

### Statistical analysis

Descriptive statistics were used, including median and interquartile range.

## Results

### Systematic review literature search results

**Fig. 1:**
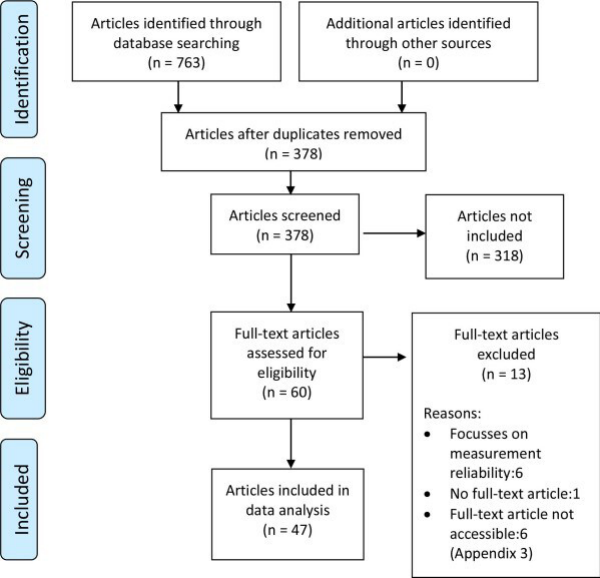
PRISMA flow diagram. A total of 763 articles were identified from the database search. Following abstract screening and full-text assessment, 47 articles remained for the final analysis.

The literature search identified 763 articles. Following study scrutinization, 47 articles remained for final analysis. The PRISMA flow diagram is presented in Figure 1. Supplementary table ii lists the papers that were accordingly excluded prior to the full-text assessment, while Supplementary table iii details the studies included in the systematic review.

### Study characteristics

There were 29 retrospective studies, nine prospective studies and nine cross-sectional studies. The most common intervention was surgery (n = 22). Other interventions included hip surveillance (n = 18) and physiotherapy (n = 1). The mean number of CP patients included per study was 207 (50 and 1,171). The mean age of study participants ranged between two and 14.6 years. Most studies had a higher proportion of males (58%; 44% to 75%). The mean duration of follow-up ranged from 1.2 and 12.8 years. A full list of study and patient characteristics for each study can be seen in Supplementary table iv.

### Measurements

In all, 14 distinct radiological measurements were reported across the 47 studies. The median (interquartile range) of measurements reported per study was two (1 to 5). Reimers’ migration percentage was the most common measurement (n = 44/47; 94%). The full list of measurements identified are presented in Table I. Clear details of the method used to calculate the measurements were not stated in most studies (n = 28/47; 60%); nevertheless, definitions were consistent across studies that did clearly provide one. Supplementary table v contains the measurements and verbatim definitions in each study.

**Table I.** List of radiological measurements identified through the systematic review, along with the total number of studies reporting each measurement. A total of 47 studies were reviewed, identifying 14 distinct measurements. Reimers’ migration percentage was the most common measurement.

Radiological measurement	Studies reporting the measurement, n
Reimers’ migration percentage	44
Neck-shaft angle	16
Acetabular index	13
Head-shaft angle	11
Centre edge angle	7
Sharp’s angle or acetabular angle	4
Acetabular depth ratio	3
Mose hip ratio	1
Shenton’s line	1
Epiphyseal shaft angle	1
Pelvic femoral angle	1
Pelvic adjusted migration percentage	1
Medialization index	1
Hilgenreiner epiphyseal angle	1

### Delphi study

### Participant characteristics

The 14 measurements identified in the systematic review were presented to 22 participants in the first round of the Delphi process, including 21 orthopaedic surgeons (95%) and one physiotherapist (5%). Participation was predominantly from the UK (n = 17; 77%), with two additional participants from the USA (n = 2; 9%), and one each from the Netherlands, India, and Thailand. Only one participant did not also take part in round two.

### Round one and two

**Fig. 2:**
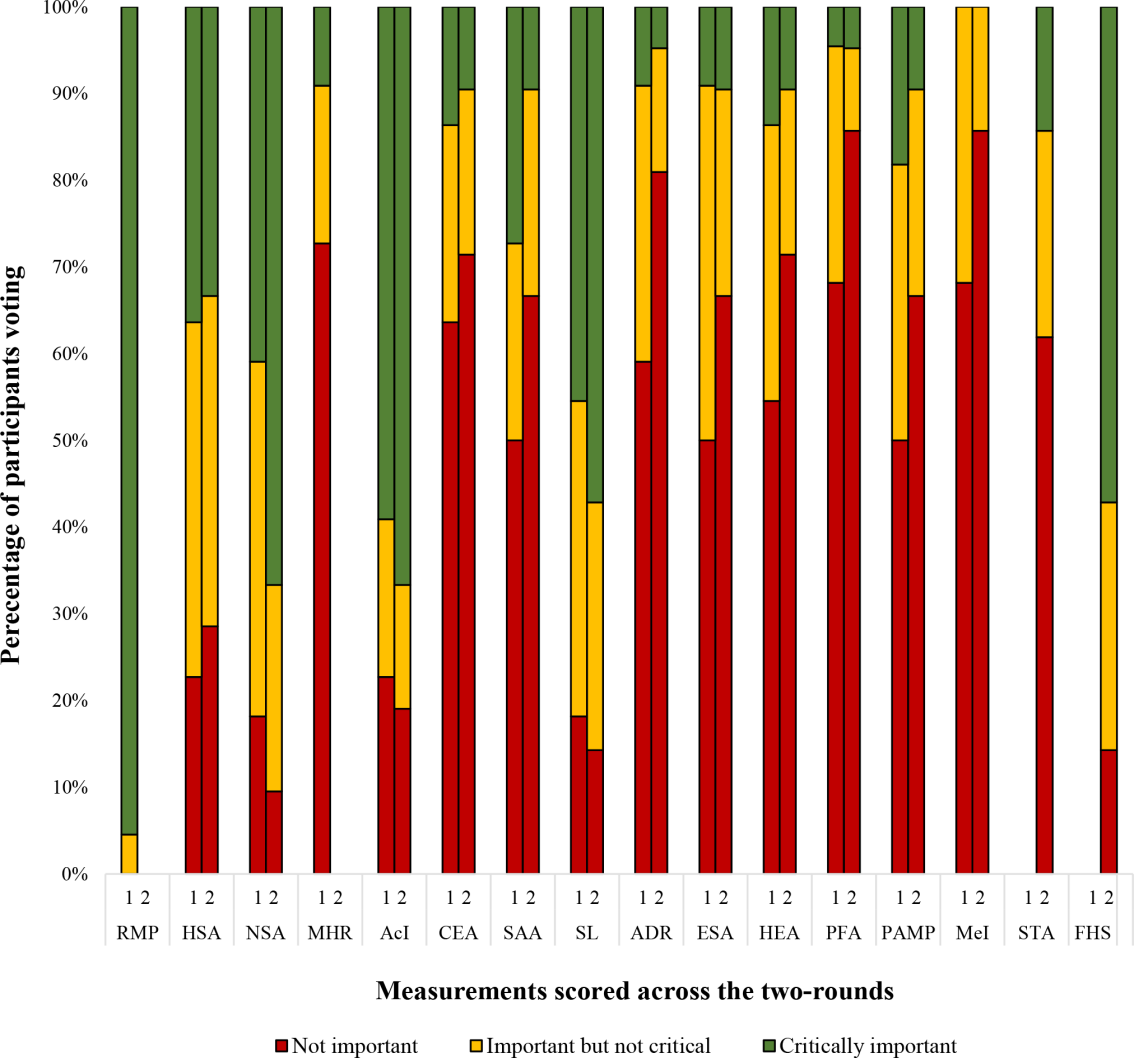
Summary of Delphi responses over round one and two. A total of 16 measurements were scored over the two rounds with Reimers’ migration percentage reaching 'consensus in' and Mose hip ratio, centre edge angle, acetabular depth ratio, Hilgenreiner epiphyseal angle, pelvic femoral angle and medialization index reaching 'consensus out'. Sourcil Tönnis angle and femoral head shape/congruency were scored for the first time in round two.

At the end of round one, two of the 14 measurements reached consensus (Figure 2). Reimers’ migration percentage was voted ‘include’, and Mose hip ratio was voted ‘exclude’. Two new measurements were also suggested: Sourcil Tönnis angle and femoral head shape/congruency; they were included in round two.

In round two, 14 measurements were presented, excluding the two that reached consensus and including the two newly suggested measurements. Of these measurements, five reached consensus to ‘exclude’ from the CMS (centre edge angle, acetabular depth ratio, Hilgenreiner epiphyseal angle, pelvic femoral angle, and medialization index); none reached consensus for ‘include’. In total, after the two rounds of Delphi, one measurement was voted ‘include’, six were ‘exclude’, and nine measurements did not reach consensus (see Supplementary table vi).

### Final consensus meeting

The nine remaining equivocal measurements were discussed in a final consensus meeting. Of these, the consensus group decided that head-shaft angle should be included in the CMS. There was a debate between head-shaft angle and neck-shaft angle, as both were broadly identified to be important throughout the Delphi study. However, neck-shaft angle is known to be poorly reproducible in CP, given the influence of hip rotation.^23^ Furthermore, the consensus group identified that head-shaft angle had greater utility as part of the risk calculation of hip displacement using the Uppföljningsprogram för cerebral pares (CPUP) Hip Score.^24^

Acetabular index was also keenly discussed as a useful measurement, but was ultimately excluded as it was not considered critically important, although potentially advantageous to record in future research. Overall, of the nine equivocal measurements, only head-shaft angle was included in the CMS.

**Fig. 3:**
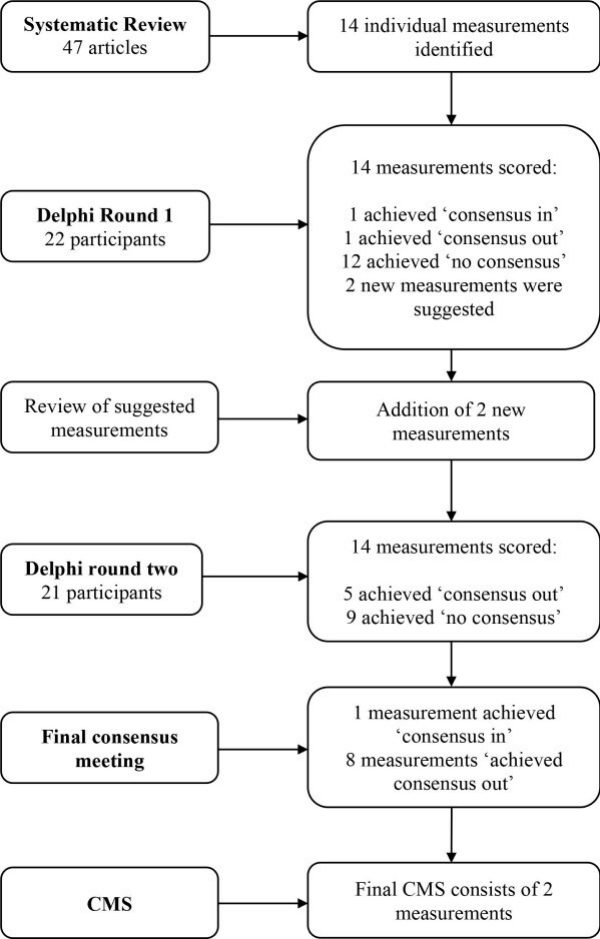
Overview of the development of the CMS. Fourteen measurements were initially identified from the systematic review. After undertaking a Delphi process, two measurements reached consensus to form the core measurements set: Reimer’s hip migration and femoral head-shaft angle.

Therefore, the final CMS consisted of Reimers’ migration percentage and head-shaft angle (Figure 3).

## Discussion

This is the first study to establish a CMS for reporting in studies on CP hip disease. Clinicians were clear that Reimers’ migration percentage was the most useful measurement, with head-shaft angle adding additional critical information to inform decision-making; therefore, they comprise the CMS.

Reimers’ migration percentage was the only measurement to reach consensus to ‘include' during the two-round Delphi survey, with 95% of participants considering it 'critically important'. This widespread consensus is reflected in the literature. Reimers’ migration percentage was the most reported measurement in the systematic review, appearing in 96% of studies. For comparison, the second most reported measurement appeared in 60% studies. Reimer’s migration percentage also has an excellent intra- and inter-rater reliability.^25-28^ Moreover, Analan et al^26^ concluded that physician experience does not affect results, further corroborating its reliability. Reimers’ migration percentage is also minimally influenced by femoral rotation and can be measured easily.^29^ Consequently, Reimers’ migration percentage is considered the gold standard for assessing hip displacement in CP.^28,30,31^

Head-shaft angle was the fourth most commonly reported measurement in the systematic review. Controversies exist regarding the utility of HSA as a predictor of hip displacement, particularly in children aged below five years.^30,32,33^ This was reflected in the Delphi responses, demonstrating ‘no consensus’ after two rounds. Despite the debate surrounding its utility, its contribution to the CPUP hip score adds to its value.^24,34,35^ The CPUP hip score has been assessed in multiple populations, achieving a high discriminatory accuracy in evaluating risk of hip displacement.^24,36^ Prospectively, the automatic calculation of core radiological measurements and hip displacement risk scores stand as an essential requirement for a fully-autonomous hip surveillance system; omitting head-shaft angle from the CMS could limit the potential application of such a system.^24^ Moreover, the head-shaft angle percentage is already standardized within the protocol of prominent national hip surveillance programmes such as CPIPS, further highlighting its utility.^14,37^ In line with these factors, head-shaft angle was deemed ‘critically important' in the final consensus meeting, warranting inclusion in the CMS.

The other measurement that caused uncertainty was neck-shaft angle. While neck-shaft angle is of clear clinical utility in planning surgery in CP,^38,39^ its role in surveillance is unclear. Neck-shaft angle requires both hips to be appropriately rotated to get an accurate measurement. Insufficient internal rotation of the hip results in inaccurate measurements.^40^ Although mathematical solutions have been developed to correct this rotational effect, the correctional outcome cannot be reliably verified.^41^Additionally, there are other shortcomings that further support its exclusion from the CMS. Boese et al^41^ reported high variance in the reporting of neck-shaft angle and identified inconsistent methods of measurement as the main concern. Inconsistently reported measurements cannot successfully facilitate comparisons between studies or achieve uniformity in the reporting of CP hip radiographs. Furthermore, some authors have identified a preference for head-shaft angle over neck-shaft angle, suggesting that neck-shaft angle may underestimate the deformity of the proximal femur given the valgus position of the femoral head in comparison to the femoral neck.^42^

The formation of a CMS will standardize the evaluation of hip radiographs in CP children and reduce heterogeneity in the reporting of CP hip radiographs. Up to now, it has been difficult to make comparisons between studies, with few studies being directly comparable. This lack of uniformity is also evident across surveillance programmes. For example, the Australian Hip Surveillance Guidelines for Children with Cerebral Palsy (2020)^43^ only supports the recording of Reimer’s migration percentage, while Scotland’s CPIPS manual (2017)^44^ additionally includes head-shaft angle. The implementation of a CMS can improve the quality of reporting, reduce the risk of reporting bias, and allow comparisons to be drawn between individual patients, centres, and studies. We hope this CMS will directly inform the development of AI tools to analyze radiographs in hip surveillance programmes. Fully-automated measurements would optimize monitoring, easing clinician work-load,^16,17^ and potentially facilitating reliable, consistent, and accurate calculations. Moreover, this software could mitigate healthcare inequalities, especially in regions with limited healthcare access where implementing the CMS may be impractical. This study identified the key measurements needed to create clinically impactful software.

The strengths of this study lie in the robust consensus building process used to establish the CMS; however, as is inherent to all consensus studies, the final output reflects the participants sampled. Our study includes a broad array of clinicians, with transparency throughout in how and why decisions were made. The biggest criticism may come from the decisions made during the final consensus meeting, though the lack of clear consensus through the Delphi meant that definitive decisions needed to be made. The Delphi survey also suffered from a lack of participation by healthcare professionals other than orthopaedic surgeons. In particular, participation from physiotherapists could have been improved given their increasing role in assessing children with CP.

In summary, we have identified the minimum measurements that should routinely be recorded and reported in studies/surveillance programmes of hip disease in children with CP. The CMS consists of two measurements: Reimers’ migration percentage and head-shaft angle. These measurements will help standardize the evaluation of CP hip disease, allowing better comparisons to be made in audit and research, as well as potentially improving surveillance programmes. We hope that this will also inform the development of software capable of automatically analyzing hip radiographs in children with CP, to enable automation and standardization.

**Take home message**

- The core measurement set (CMS) for analyzing hip disease among children with cerebral palsy includes Reimers' migration percentage and femoral head-shaft angle.

- Implementing this CMS can promote consistency, facilitate comparisons, and support the development of tools to automate analysis in clinical practice, research, and hip surveillance programmes.
